# Antiviral Properties of HIV-1 Capsid Inhibitor GSK878

**DOI:** 10.1128/aac.01694-22

**Published:** 2023-04-11

**Authors:** Chunfu Wang, Haichang Huang, Kirsten Mallon, Lourdes Valera, Kyle Parcella, Mark I. Cockett, John F. Kadow, Eric P. Gillis, Mark Krystal, Robert A. Fridell

**Affiliations:** a Discovery Biology, ViiV Healthcare, Branford, Connecticut, USA; b Discovery Chemistry, ViiV Healthcare, Branford, Connecticut, USA

**Keywords:** capsid inhibitor, antiviral activity, capsid core stability

## Abstract

GSK878 is a newly described HIV-1 inhibitor that binds to the mature capsid (CA) hexamer in a pocket originally identified as the binding site of the well-studied CA inhibitor PF-74. Here, we show that GSK878 is highly potent, inhibiting an HIV-1 reporter virus in MT-2 cells with a mean 50% effective concentration (EC_50_) of 39 pM and inhibiting a panel of 48 chimeric viruses containing diverse CA sequences with a mean EC_50_ of 94 pM. CA mutations associated with reduced susceptibility to other inhibitors that bind to PF-74 binding site (L56I, M66I, Q67H, N74D, T107N, and Q67H/N74D) also reduced susceptibility to GSK878, with M66I, Q67H/N74D, and L56I having the greatest impact on antiviral activity. Amino acid substitutions in the CA cyclophilin A (CypA) binding loop (H87P and P90A), distal from the inhibitor binding site and associated with reduced CA-CypA binding, subtly, but reproducibly, also decreased GSK878 potency. Mechanism-of-action studies showed that GSK878 blocked both early (preintegration) and late (postintegration) steps in HIV-1 replication, with the early inhibition primarily determining the compound’s antiviral activity. The early inhibition results from blocks to HIV-1 nuclear import and proviral integration and is associated with altered stability of the HIV-1 CA core.

## INTRODUCTION

HIV-1 capsid (CA) is a highly conserved and essential viral protein that plays multiple roles in HIV-1 replication (1–3). The CA monomer is a 231-amino-acid protein with distinct amino-terminal (NTD) and carboxy-terminal (CTD) structural domains separated by a flexible linker region (4–8). Prior to proteolytic cleavage, CA domain interactions of the Gag p55 polyprotein drive the assembly of immature virions in infected cells and stabilize the hexameric Gag lattice that forms the spherical structural shell of immature virions (9–11). Following virion budding and proteolytic cleavage, CA monomers released from the Gag p55 precursor reassemble into the mature HIV-1 core, which encapsulates the RNA genome and replicative enzymes of infectious virus (11–13). The HIV-1 core is a fullerene cone composed of ~200 to 250 CA hexamers and 12 CA pentamers (14–16). Early in the infection process, the CA core interacts with multiple cellular proteins and mediates transportation of the HIV-1 ribonucleoprotein complex through the cytoplasm to the nuclear pore complex (NPC) and into the host cell nucleus (2, 3, 17–19). Recent evidence suggests that the HIV-1 core remains largely intact in the host cell nucleus and that CA uncoating and release of the viral preintegration complex occur near the site of integration of the HIV-1 cDNA into the host cell genome (20–25). In addition, efficient reverse transcription, which likely initiates in the cytoplasm but completes in the nucleus, appears to require the presence of at least a partial CA hexamer lattice (22, 24, 26, 27).

Given its multiple essential roles in HIV-1 replication and its high degree of conservation, HIV-1 CA is an attractive target for therapeutic intervention (28–32). Although several inhibitor binding sites on CA have been identified (33), the site first identified as the binding site of PF-3450074 (PF-74) has emerged as the most promising pharmacological target (30, 31, 34–38). Clinical proof of concept for this site has been provided by lenacapavir (LEN), a compound that has shown promising antiviral activity in both naive and heavily treatment-experienced (HTE) HIV-1-infected individuals and was approved in Europe in the HTE subpopulation as an every-6-month subcutaneous dosing regime on 17 August 2022 (39–41; https://www.ema.europa.eu/en/medicines/human/EPAR/sunlenca). LEN was also approved by the U.S. FDA on 22 December 2022 for treatment of the HTE HIV-1 patients as a 6-month subcutaneous dosing regime (36–38; https://www.fda.gov/news-events/press-announcements/fda-approves-new-hiv-drug-adults-limited-treatment-options). LEN is currently also being developed as an oral and long-acting subcutaneous injection for naive treatment and other HIV-1-infected subpopulations (39, 41). In this study, we examined the antiviral properties of a recently disclosed CA inhibitor, GSK878, that binds the same pocket on the mature CA hexamer as PF-74 and LEN (42). We show that GSK878 is an effective HIV-1 replication inhibitor in cell culture with potent antiviral activity against a range of HIV-1 strains and a wide spectrum of CA sequences. GSK878 blocks both early (preintegration) and late (postintegration) steps in the HIV-1 replication cycle; however, its antiviral potency appears to be primarily derived from disrupting nuclear entry and altering CA core stability, thereby preventing HIV-1 integration.

## RESULTS

**FIG 1:**
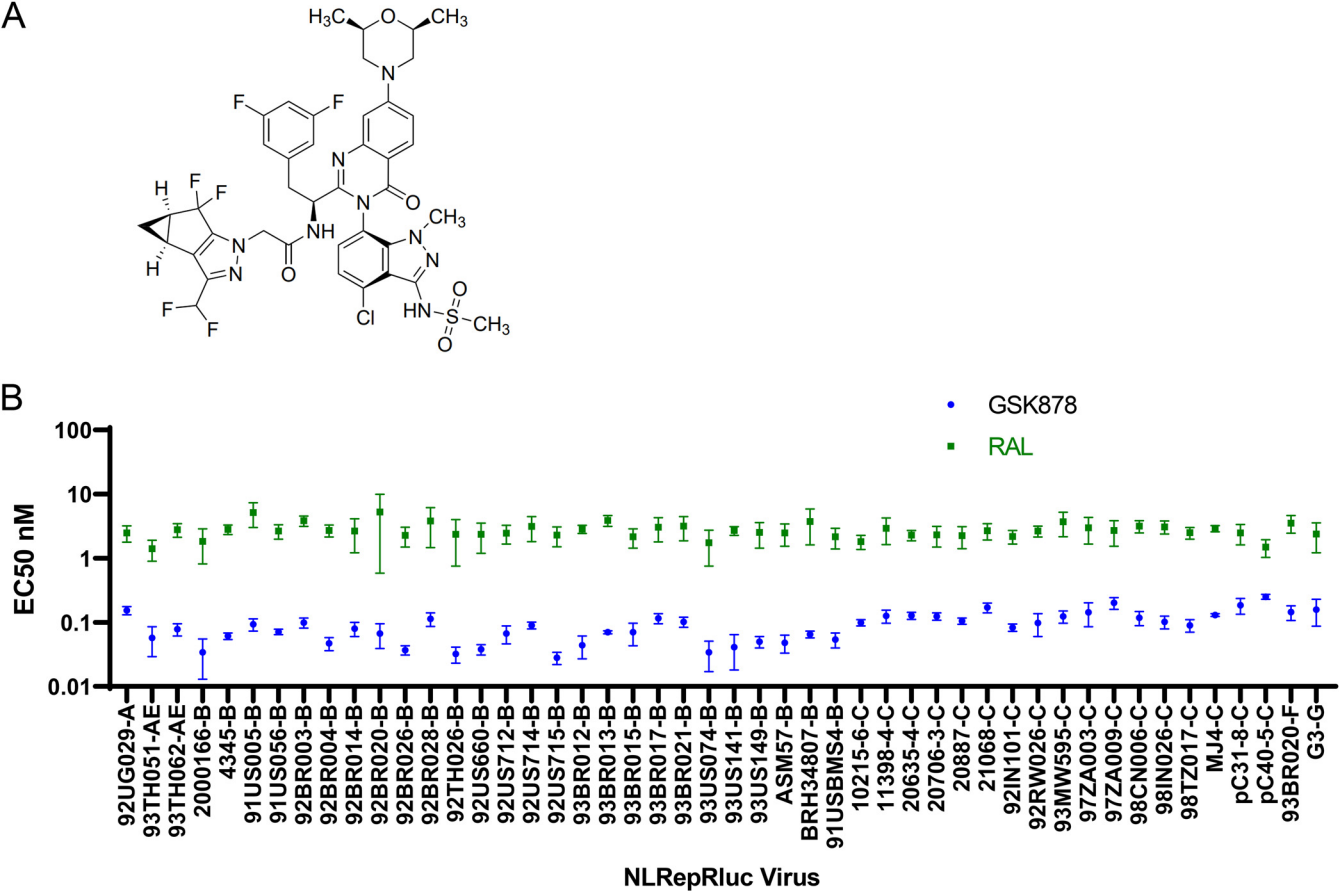
(A) Chemical structure of GSK878. (B) Susceptibility of chimeric reporter viruses to GSK878 and RAL. Shown is a plot of EC_50_ values calculated from antiviral assays performed in MT-2 cells with replication-competent reporter viruses (NLRepRluc) with the Gag-PR region derived from HIV-1 clinical isolates of the indicated strain and subtype. Values are means and SD from ≥4 experiments.

GSK878 is a newly reported, potent HIV-1 CA inhibitor (42). The structure is shown in Fig. 1A. Its synthesis and characterization have been described in detail by Gillis et al. (42). The antiviral activity of GSK878 toward an NL_4-3_-based replication-competent HIV-1 reporter virus (NLRepRluc-WT) was assessed in MT-2 cells. GSK878 inhibited the reporter virus with a mean 50% effective concentration (EC_50_) of 0.039 ± 0.014 nM and a mean 90% effective concentration (EC_90_) of 0.101 ± 0.028 nM (92 independent values). The 50% cytotoxicity concentration (CC_50_) was greater than 20 μM (8 independent values), yielding a therapeutic index (TI; CC_50_/EC_50_) of >512,820. Precipitation of the compound in the cell culture medium prevented cytotoxicity determination at concentrations exceeding 20 μM. NLRepRluc-WT is derived from the NL_4-3_ strain and has a *Renilla* luciferase (Rluc) reporter gene inserted in place of Nef coding sequences (43). To determine the potency of GSK878 toward nonreporter HIV-1 strains, the susceptibilities of 7 full-length HIV-1 strains were examined in the Rev-dependent A3R5 indicator T cell line (44). GSK878 inhibited these full-length viruses with EC_50_ values ranging from 0.022 to 0.216 nM, with a mean EC_50_ value of 0.081 ± 0.068 (Table 1). In comparison, the nonnucleoside reverse transcriptase inhibitor (NNRTI) efavirenz (EFV) inhibited the viruses with a mean EC_50_ value of 0.610 nM ± 0.360 (EC_50_ values ranging from 0.350 to 1.38 nM) (Table 1). To further assess the ability of GSK878 to inhibit viruses with diverse CA sequences, drug susceptibility assays were performed in MT-2 cells with a panel of 48 recombinant NLRepRluc viruses with Gag protease (Pr)-encoding sequences derived from HIV-1 clinical isolates (45) (Fig. 1B; see also Table S1 in the supplemental material). The virus panel, containing representatives from HIV-1 clades A, B, C, F, G, and CRF_AE, was inhibited with a mean EC_50_ of 0.094 ± 0.049 nM and values ranging from 0.028 to 0.251 nM. A reference integrase strand transfer inhibitor (INSTI), raltegravir (RAL), inhibited the panel of chimeric viruses with a mean EC_50_ value of 2.77 ± 0.77 nM and values ranging from 1.41 to 5.24 nM. Since integrase sequences in the chimeric viruses were identical (derived from the parental NLRepRluc virus), the variation in EC_50_ values observed with RAL (3.7-fold; 5.24/1.41) likely reflects normal assay variation, possibly including differences in replicative abilities of the chimeric viruses.

**TABLE 1:** Susceptibilities of laboratory-adapted HIV-1 strains to GSK878

Virus	GSK878		EFV^*a*^	
	Mean EC_50_ ± SD, nM	*n* ^*b*^	Mean EC_50_ ± SD, nM	*n*
NL_4-3_	0.052 (0.060, 0.044)	2	0.359 (0.372, 0.347)	2
BaL	0.022 ± 0.005	4	0.665 ± 0.168	4
HXB2	0.033 ± 0.007	4	0.514 ± 0.171	4
IIIB	0.049 ± 0.009	4	0.592 ± 0.148	4
LAI	0.068 ± 0.041	4	0.350 ± 0.186	4
MN	0.124 ± 0.028	3	0.409 ± 0.131	4
RF	0.216 ± 0.082	4	1.38 ± 0.498	4
Mean	0.081 ± 0.068	7	0.610 ± 0.360	7

a Efavirenz (EFV) was used as a reference.

b *n*, number of independent values.

GSK878 binds in a pocket formed by adjacent CA monomers in the context of the mature CA hexamer (42). This is the pocket previously identified as the binding site of PF-74, GS-CA1, and LEN (GS-6207) (41, 46–50). Several CA amino acid substitutions that alter the potency of these inhibitors have been identified in resistance selection studies with these compounds (40, 41, 48, 50). A subset of these substitutions (L56I, M66I, Q67H, K70R, N74D, H87P, T107N, L111I, and Q67H/N74D) was introduced into the NLRepRluc backbone as amino acid changes to assess their effect on susceptibility to GSK878 in MT-2 cells (Table 2). Of these, the L56I, M66I, and N74D single substitutions and the Q67H/N74D double substitution markedly reduced susceptibility to the compound (~67- to 25,042-fold relative to that of the wild-type [WT] virus), while the Q67H and T107N single substitutions moderately reduced susceptibility (~6- to 10-fold). The remaining changes (K70R, H87P, and L111I) had lesser effects on GSK878 potency (<3-fold). Similar results have been reported with PF-74 and LEN, suggesting similar binding modes of these compounds (40, 41, 51, 52).

**TABLE 2:** Effects of site-directed CA mutations on GSK878 antiviral activity

Variant	GSK878		RAL^*a*^	
	EC_50_ fold change relative to wild type^*b*^	*n*	EC_50_ fold change relative to wild type^*b*^	*n*
L56I	2,081.5	4	0.6	8
M66I	25,042.4	4	0.6	8
Q67H	6.4	4	0.5	6
K70R	2.5	4	0.8	8
N74D	67.1	4	0.7	6
H87P	2.1	72	1.0	49
T107N	9.8	4	1.0	6
L111I	1.7	4	0.8	6
Q67H/N74D	3,096.2	6	0.6	14

a RAL, raltegravir.

b Assay with NLRepRluc viruses in MT-2 cells.

**FIG 2:**
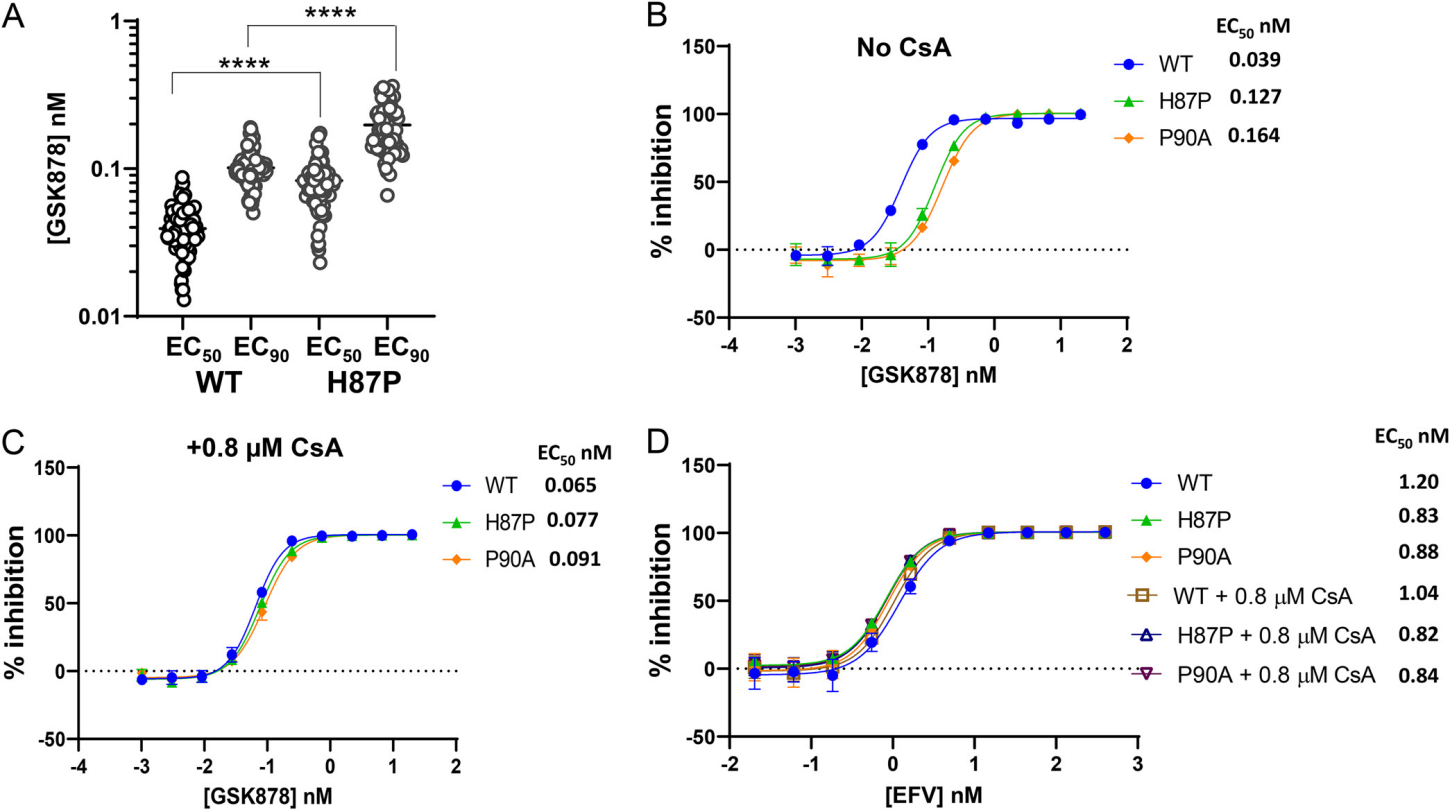
Effect of cyclophilin A on GSK878 antiviral activity. (A) Scatterplot of EC_50_ and EC_90_ values of GSK878 from antiviral assays with wild-type (WT) and CA H87P HIV-1 reporter viruses (NLRepRluc) in MT-2 cells (****, *P* < 0.0001; WT, *n* = 92; H87P, *n* = 72). (B and C) Comparison of GSK878 dose-response inhibition curves from antiviral assays in MT-2 cells with VSV-G-pseudotyped replication-defective HIV-1 (VSV-G:NLRepRlucΔENV) with the indicated CA missense mutations in the absence (B) or presence (C) of 0.8 μM CsA. Points are averages of two experiments, each performed in duplicate. (D) Dose-response curves with a reference NNRTI (EFV) under the same conditions as for panels B and C.

Most of the amino acid substitutions tested, including those that had the largest effect on susceptibility to GSK878, are located within or near the inhibitor binding site (41, 42, 46, 49, 50, 52). In contrast, H87P, identified in a resistance selection study with an analog of PF-74 (48), resides within the CA cyclophilin A (CypA) binding loop, distal to the inhibitor binding pocket (5, 46). In multiple experiments, the H87P substitution was associated with a small (2.1-fold) but statistically significant reduction in susceptibility to GSK878 (Fig. 2A). Similar results were obtained with the reference CA inhibitor, LEN (Fig. S1A). Given the location of the H87P substitution, these results suggested the possibility that CypA binding to CA might influence the potency of the CA inhibitors. To test this possibility, the antiviral activity of GSK878 in MT-2 cells was assessed in single-cycle infection assays with replication-defective viruses (NLRepRlucΔENV) pseudotyped with vesicular stomatitis virus glycoprotein (VSV-G). In addition to the parental (WT) and H87P viruses, a virus with a P90A amino acid substitution, a well-characterized CA mutation that disrupts the CA-CypA interaction (53–55), was included in the analysis (Fig. 2). In this assay format, the effects of the H87P and P90A substitutions on GSK878 potency were similar, reducing susceptibility ~3.3- and 4.2-fold, respectively (Fig. 2B). However, when the CA-CypA interaction was pharmacologically blocked by treatment with 0.8 μM CsA, no difference in the susceptibilities of the WT, H87P, and P90A viruses to GSK878 was observed (Fig. 2C), confirming that CypA binding to CA has the ability to alter inhibitor potency, albeit modestly. Similar results with GSK878 were obtained in assays performed in the presence of 1.2 μM CsA (Fig. S1B and C). The potencies of reference NNRTI (EFV) and INSTI (RAL) compounds were largely unaffected by the H87P and P90A substitutions or by the addition of CsA (Fig. 2D and Fig. S1D), confirming that the results were specific for the CA inhibitor.

PF-74, GS-CA1, and LEN, compounds that bind the same site on the mature CA hexamer as GSK878, possess both early (preintegration) and late (postintegration) anti-HIV-1 activities (41, 46, 48, 50). As shown in Table 3, GSK878 inhibited both early and late HIV-1 replication steps as well. The late inhibition was associated with a decrease in virus production from infected cells as measured by p24 release (Table 3). However, the early inhibitory activity of GSK878 was ~30-fold more potent than the late activity, indicating that the antiviral potency of the compound primarily derives from inhibition of an early replication step. Control compounds for late activity (nelfinavir [NFV], a protease inhibitor) and early activity (RAL, an INSTI) exhibited the expected antiviral profiles in the assay (Table 3).

**TABLE 3:** Inhibition profile of GSK878 in early and late HIV-1 replication steps

Drug	EC_50_ ± SD, nM^*a*^		IC_50_ ± SD, nM,^*a*^ p24
	Early	Late	
GSK878	0.063 ± 0.021	1.90 ± 0.23	3.24 ± 1.45
RAL	3.19 ± 2.24	>300	>200
NFV	>1,000	16.29 ± 2.20	ND

a Means and standard deviations from 3 experiments. ND, not done; NFV, nelfinavir.

**FIG 3:**
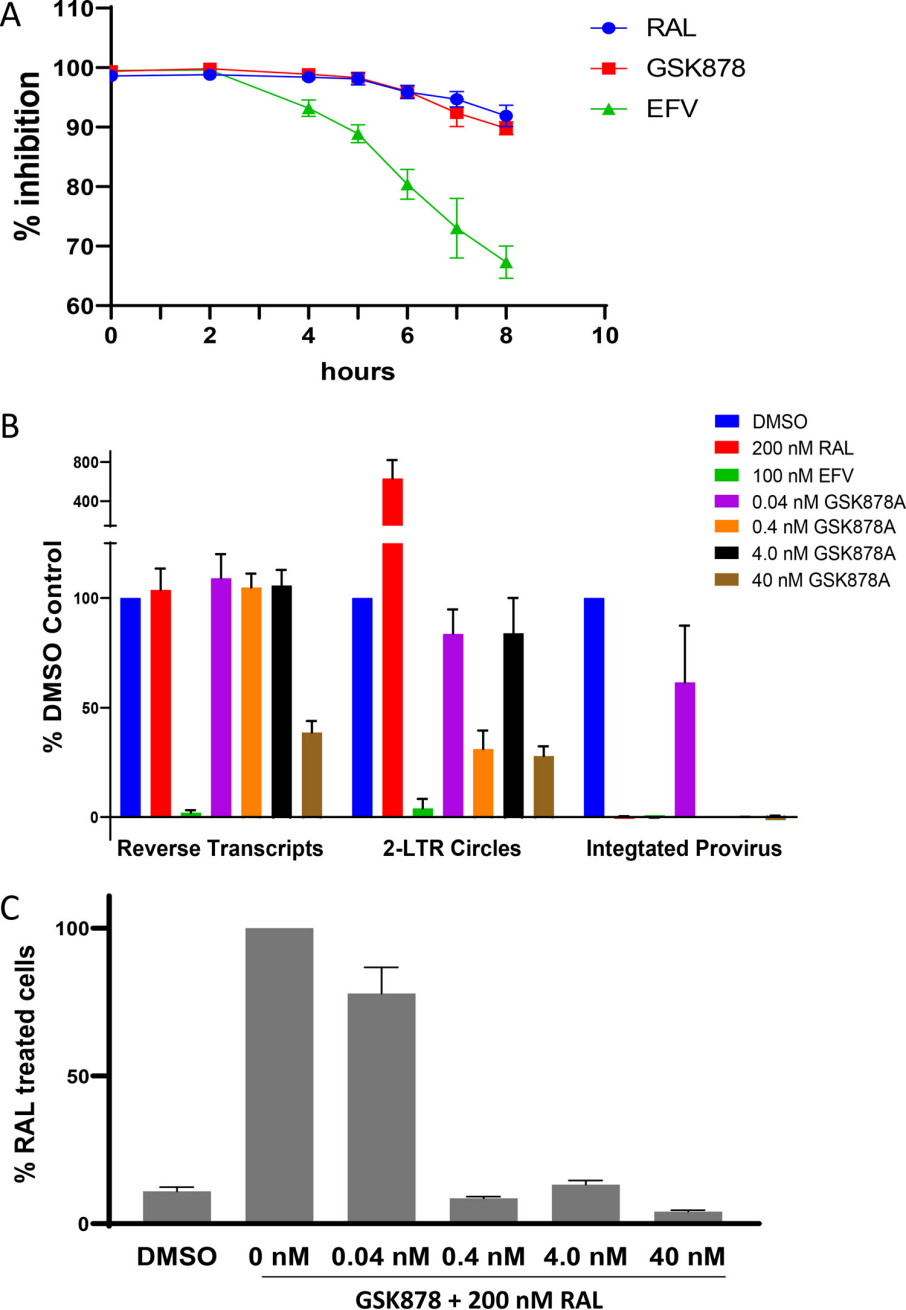
GSK878 antiviral activity. (A) Time-of-addition experiment. MT-2 cells were infected with a replication-defective virus pseudotyped with gp160 from HIV-1 strain LAI (LAI:NLRepRlucΔEnv). Inhibitors were added at 0, 2, 4, 5, 6, 7, and 8 h postinfection, and *Renilla* luciferase activity was measured after an additional 72 h. Percent inhibition relative to the value for DMSO-treated control cells is plotted for each time point (means and SD from 8 replicates). (B) Effect of GSK878 on HIV-1 replication intermediates. MT-2 cells infected with replication-defective VSV-G-pseudotyped HIV-1 (VSV-G:NLRepRlucΔENV) were treated with the indicated inhibitors and harvested at 12, 24, and 48 h for quantification of total reverse transcripts, 2-LTR circles, and integrated proviruses, respectively (means and SD from ≥3 experiments). (C) Quantification of 2-LTR circles from VSV-G:NLRepRlucΔENV-infected cells treated with the indicated concentration of GSK878 in the presence of 200 nM RAL.

To better understand which early HIV-1 replication step is inhibited by GSK878, a time-of-addition assay was performed. In this assay, inhibitors were added at set time points after infection of MT-2 cells with a replication-defective reporter virus pseudotyped with HIV-1 LAI gp160. As shown in Fig. 3A, a reference NNRTI (EFV) began to lose inhibitory activity by 4 h postinfection, with further reductions in activity observed at later time points. In contrast, GSK878 and an INSTI (RAL) only began to show a slight reduction in inhibitory activity at 6 h postinfection. In fact, the inhibition profiles of GSK878 and RAL were very similar (Fig. 3A), suggesting that GSK878 inhibits a step that occurs after reverse transcription and near the time of integration.

The early inhibitory activity of GSK878 was further examined with quantitative PCR (qPCR) assays targeting HIV-1 replication intermediates (56) (Fig. 3B). In these assays, the accumulation of late reverse transcripts, 2-long terminal repeat (LTR) circles, and integrated provirus was measured in the presence of GSK878 at concentrations roughly correlating to 1× (0.04 nM), 10× (0.4 nM), 100× (4.0 nM), and 1,000× (40 nM) EC_50_ of the inhibitor as measured in the VSV-G:NLRepRlucΔEnv-WT virus/MT-2 cell assay. The most pronounced effect of GSK878 was on the accumulation of integrated provirus, which was effectively blocked at concentrations of 0.4 nM and higher. GSK878 had no effect on reverse transcripts at concentrations up to 4 nM, while at 40 nM, reverse transcripts were reduced by ~61%. The effect on 2-LTR circles, which have frequently been used as a surrogate for nuclear import of the HIV-1 nucleoprotein complex (57, 58), was more nuanced. At 0.4 nM GSK878, 2-LTR circles were reduced by ~69% compared to the no-inhibitor control, but at 4 nM, little effect on 2-LTR circles was observed. At 40 nM GSK878, 2-LTR circles were reduced by approximately 72%, consistent with the block to reverse transcripts observed at this concentration. When integration is blocked by an INSTI, an increase in 2-LTR circles is often observed, possibly reflecting an increase in substrate available for 2-LTR circle formation (56, 59). In our experiments, treatment with RAL resulted in a marked increase in 2-LTR circles compared to that in dimethyl sulfoxide (DMSO)-treated control cells (Fig. 3B). We surmised that the effect of GSK878 on nuclear import as measured by 2-LTR circles might be masked by an increase in 2-LTR circle formation due to a block in integration, similar to the increase observed upon treatment with RAL. To test this possibility, the effect of GSK878 on 2-LTR circles was assessed under conditions in which integration was completely blocked by cotreatment with 200 nM RAL. We reasoned that a reduction in 2-LTR circles under these conditions would reveal indicate a block in nuclear import of the HIV-1 cDNA. In fact, treatment with ≥0.4 nM GSK878 resulted in substantial decreases in 2-LTR circles relative to the amount observed in the presence of cells treated with RAL only (~87% to 96% [Fig. 3C]), indicating a strong, but incomplete, block by GSK878 to nuclear import of the HIV-1 nucleoprotein complex.

**FIG 4:**
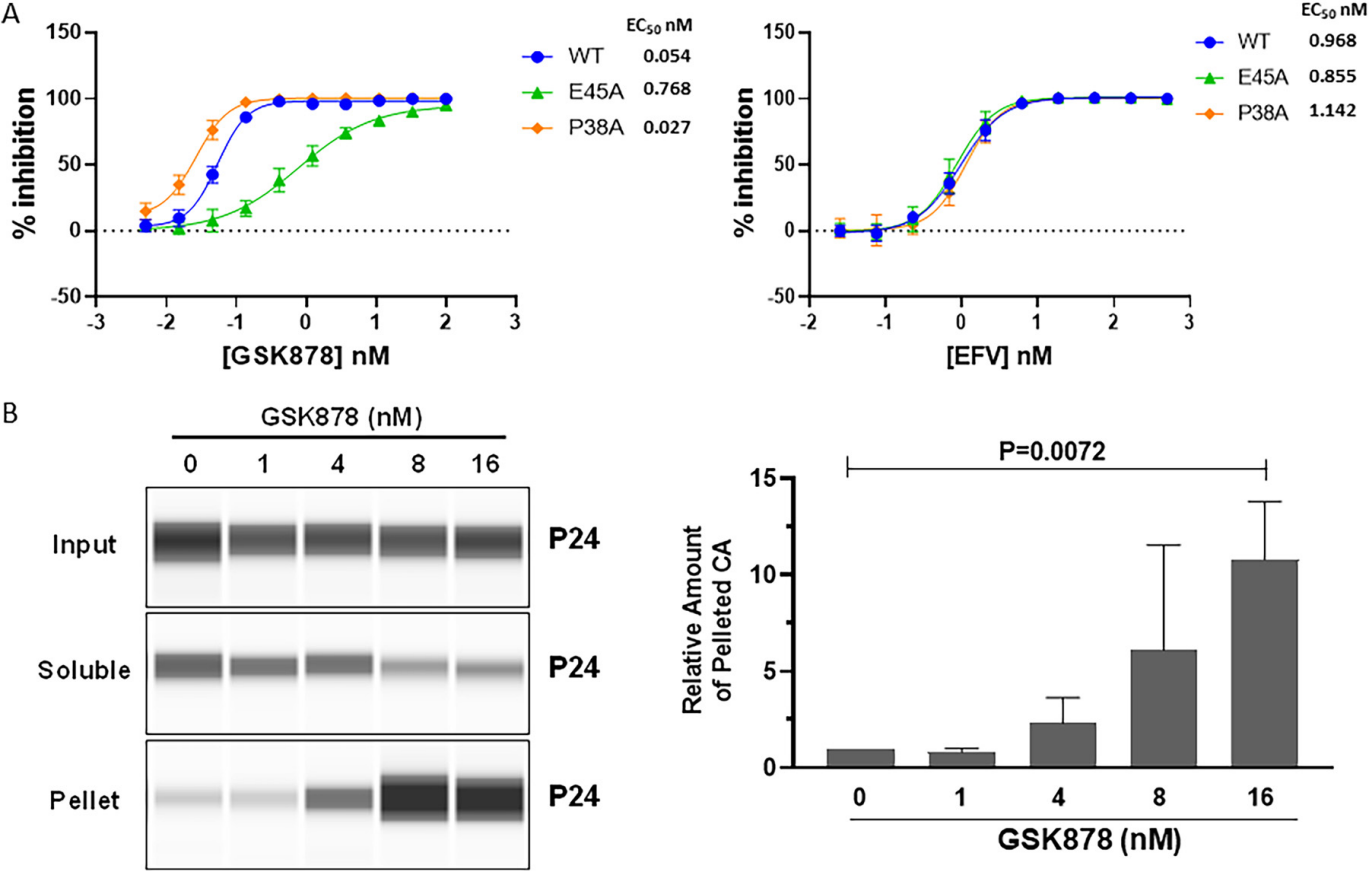
GSK878 antiviral activity and CA core stability. (A) Susceptibility hyperstable (E45A) and hypostable (P38A) HIV-1 CA core mutants on GSK878 and EFV antiviral activity. Shown is a comparison of dose-response inhibition curves from antiviral assays in MT-2 cells with VSV-G-pseudotyped replication-defective HIV-1 (VSV-G:NLRepRlucΔENV), parental (WT) or with the indicated CA missense mutation (means ± SD from 3 experiments). (B) GSK878 stabilizes the CA core in infected cells. HeLa cells infected with VSV-G:NLRepRlucΔENV were treated with the indicated concentrations of GSK878 or DMSO. After 6 h, cells were harvested and analyzed by the fate-of-the-capsid assay as described in Materials and Methods. CA (p24) in the input, supernatant, and pellet was detected with the Simple Western system (Wes; Bio-Techne, Minneapolis, MN). Quantification of CA in the pellet is shown as the means ± SD from 4 experiments. The images on the left side of panel B were derived from the same blot and spliced for labeling purpose.

Several CA mutations that alter stability of the HIV-1 CA core have been identified, including the hypostable P38A mutant and the hyperstable E45A mutant (60). The potential effect of core stability on GSK878 antiviral activity was assessed with single-cycle pseudovirus assays with viruses carrying these amino acid substitutions (Fig. 4A). The P38A virus appeared slightly more sensitive to GSK878 (~2-fold), while the E45A virus was ~14-fold less sensitive to the CA inhibitor; this effect was not observed with the NNRTI (EFV), suggesting a potential effect of intrinsic CA core stability on GSK878 potency. The effect of GSK878 on core stability was next examined with a fate-of-the-capsid assay (61, 62) in HeLa cells. In this assay, which measures the stability of HIV-1 cores in cells, treatment with increasing concentrations of GSK878 led to marked increases in the amount of pelletable CA, indicating a stabilizing effect of the inhibitor on the HIV-1 CA core (Fig. 4B).

## DISCUSSION

GSK878 is a newly described HIV-1 CA inhibitor that binds the pocket formed at the junction of CA monomers of the mature CA hexamer, a site first identified as the binding site of PF-74 and later of LEN (41, 42, 47, 49). Here, we show that GSK878 is highly potent against a range of HIV-1 laboratory strains (mean EC_50_ = 81 pM) as well as against a panel of 48 chimeric reporter viruses with Gag Pr sequences derived from diverse clinical isolates (mean EC_50_ = 94 pM). Viruses with amino acid mutations associated with reduced susceptibility to LEN (L56I, M66I, Q67H, N74D, T107N, and Q67H/N74D) also showed reduced susceptibility to GSK878. We found that like PF-74 and LEN (41, 46, 48), GSK878 possesses antiviral activities targeting both early and late HIV-1 replication steps. The late antiviral activity of the compound is associated with reduced virus production from infected cells. While the full binding site of the compound between adjacent monomers on the mature CA hexamer would not be present on the Gag polyprotein, the compound could potentially disrupt virus production by binding to the CA domain of Gag, likely via interactions with the amino-terminal CA region, where extensive contacts are observed in the GSK878:CA-hexamer X-ray cocrystal (42). The late antiviral activity of GSK878 is substantially weaker than the early activity (~30-fold), indicating that the early activity drives the antiviral potency of the compound.

In the initial characterization of PF-74, a highly resistant virus with 5 mutations in CA (Q67H, K70R, H87P, T107N, and L111I) was identified (48). Of these, H87P is unique in that it is located in the CA CypA binding domain, distal from the inhibitor binding site (51). CypA binds to the HIV-1 CA core in the cytoplasm of newly infected cells, where it blocks potent TRIM-5 restriction in primary blood cells (63, 64). In addition to this key activity, CypA has also been implicated in modulating HIV-1 infectivity by influencing HIV-1 nuclear import pathways and altering CA core stability (65–67). Moreover, a functional interplay appears to exist between mutations that alter the CypA-CA interaction and mutations within the GSK878 binding pocket (e.g., P90A and N74D) (68–72). We found that a virus with an H87P substitution was slightly (~2.1-fold) but significantly (*P* > 0.0001) less susceptible to both GSK878 and LEN than the parental virus in MT-2 cells. A virus with a P90A substitution, which abrogates the CA-CypA interaction, exhibited the same sensitivity to GSK878 as H87P, suggesting that H87P also reduces CypA binding to CA. When the CA-CypA interaction was blocked with cyclosporine (CsA) treatment, the sensitivities of the WT, H87P, and P90A viruses to GSK878 were essentially the same, confirming that CypA binding influences inhibitor potency. A similar effect of CypA on PF-74 potency has been also described previously (73). Given the proposed roles of CypA in mediating CA core stability and nuclear import pathway selection, it seems likely that one of these activities accounts for the observed effect on CA inhibitors.

GSK878’s inhibition profile in a time-of-addition assay was distinct from that of an RT inhibitor and very similar to that of an INSTI, suggesting that the inhibitor blocks a step that occurs after reverse transcription and near the time of integration. Experiments monitoring HIV-1 DNA replication intermediates showed that at very high concentrations (40 nM; ~1,000× EC_50_), GSK878 partially inhibited reverse transcription. Similar blocks to reverse transcription at high inhibitor concentrations have been reported for PF-74 and LEN and may reflect disruption of the CA core at these concentrations (46, 49, 73, 74). In contrast, no effect on RT was observed at lower concentrations, where, instead, reductions were observed on the accumulation of 2-LTR circles (surrogate for nuclear import) and, most prominently, on integrated provirus. The effect on 2-LTR circles was most clear when cells were cotreated with GSK878 and a high concentration of an INSTI (RAL). Under these conditions, in which an increase in 2-LTR circles relative to those for untreated cells was observed due to the block of integration by RAL, GSK878 inhibited 2-LTR circles by ≥86% at concentrations ranging from 0.4 nM to 40 nM. These results suggest that GSK878 inhibits nuclear import and trafficking of the HIV-1 replication complex, likely by competitively blocking the binding of cell factors whose binding sites overlap the inhibitor binding site (for example, CPSF6, Nup153, and Sec24C) (49, 75, 76) and/or by altering CA core stability. However, the results also suggest that the disruption of nuclear import by itself was not enough to fully explain the antiviral activity of the compound, since the strongest inhibition was observed at the level of accumulation of integrated provirus, which was effectively blocked at inhibitor concentrations of 0.4 nM and higher. In agreement with the time-of-addition results, the effect on integration suggests an inhibitory activity of GSK878 that occurs somewhere between nuclear entry and integration. As has been proposed for LEN (46), this inhibitory activity might be explained by an effect on CA core stability, altering the timing of release of the HIV-1 preintegration complex and thereby disrupting integration. Overall, the inhibitory profile of GSK878 in these assays appears to closely resemble that described for virus like particles containing CA cores hyperstabilized by the introduction of cysteine residues (E180C and E14C/E45C) (77). These hyperstable core mutants were able to complete reverse transcription, displayed an ~5-fold decrease in 2-LTR circles, and were almost completely blocked at the integration step. The similar phenotype observed with GSK878 treatment suggests the possibility that GSK878 acts, at least in part, by stabilizing the CA core.

We assessed the potential effect GSK878 on core stability indirectly by examining the sensitivity of viruses with either hyperstable (E45A) or hypostable (P38A) CA cores (60, 78) and directly with fate-of-the-capsid assays. While the P38A hypostable core mutant had a similar, or slightly increased (~2-fold), sensitivity to GSK878 compared to the parental virus, the E45A mutant displayed substantially reduced sensitivity (~14-fold). The E45A substitution has also been shown to reduce susceptibility to PF-74 and LEN (46, 73). On the surface, this would appear to indicate that these CA inhibitors act by destabilizing the CA core, since it is reasonable to assume that higher inhibitor concentrations would be required to destabilize a more intrinsically stable core. However, multiple phenotypes have been attributed to the E45A mutant, including abrogating the ability to infect nondividing cells and altering the utilization of cell factors implicated in HIV-1 nuclear import (for example, TNPO3 and Nup153) (69, 79–81). Moreover, a second site mutation (R132T) was found to rescue the ability of an E45A mutant to infect nondividing cells without altering the stabilizing effect of E45A on the CA core (82). Therefore, it is difficult to know if the reduced susceptibility to GSK878 observed with E45A is due to hyperstabilization of the core or if other factors, such as the impact of the mutation on nuclear import, might also be responsible. As was previously shown with LEN (83), treatment with GSK878 resulted in a clear increase in pelletable CA in fate-of-the-capsid assays, indicating a stabilizing effect of the compound on the CA core. Overall, these results are consistent with GSK878 inhibiting the early steps of HIV-1 replication by two primary mechanisms: (i) inhibiting nuclear entry of the HIV-1 nucleoprotein complex, possibly by competing for CA binding with host cofactors, and (ii) enhancing the stability of the CA core, thereby altering timely release of the preintegration complex resulting in a block to integration. In addition, GSK878 was recently shown to direct HIV-1 integration away from active transcription units into what appears to be a more random pattern of integration throughout the host cell genome, a phenotype it shares with LEN (F. Bushman, personal communication). While further development of GSK878 was not pursued due to an unfavorable preclinical toxicology finding, lessons learned from evaluation of GSK878 and other analogs have been important in advancing CA inhibitors to clinical development.

## MATERIALS AND METHODS

### Vectors, viruses, cells, and compounds.

HEK-293T and HeLa cells were obtained from the American Type Culture Collection (ATCC; Manassas, VA). Rev-responsive A3R5-GFP/Luc cells (44) were purchased from 101Bio (Mountain View, CA). MT-2 cells were obtained from the NIH AIDS Reagent Program (Manassas, VA). B6 cells, MT-4-derived cells stably transduced with a firefly luciferase reporter gene under the transcriptional control of the HIV-1 LTR, have been described previously (45). MT-2 and B6 cells were propagated in RPMI 1640 medium supplemented with 10% heat-inactivated fetal bovine serum (FBS), 10 mM HEPES buffer (pH 7.5), 2 mM l-glutamine, 100 U/mL of penicillin G, and 100 μg/mL of streptomycin. A3R5-GFP/Luc cells were propagated in the same medium supplemented with 0.5 mg/mL of G418 and 1 μg/mL of puromycin. HEK-293T and HeLa cells were propagated in Dulbecco’s modified Eagle medium (DMEM) containing 10% FBS, 100 U/mL of penicillin G, and 100 μg/mL of streptomycin. The reporter virus DNA clone, pNLRepRluc (pNLRepRluc-WT), which has a *Renilla* luciferase gene in place of *nef* sequences, has been described previously (43). Site-directed mutations in CA were introduced with standard recombinant PCR techniques and verified by sequence analysis. A panel of chimeric pNLRepRluc proviral clones with the Gag Pr-encoding region derived from HIV-1 clinical isolates has been described previously (45). pNLRepRlucΔEnv, with a deletion encompassing gp160 amino acids 133 to 202, was constructed by standard molecular biology techniques from pNLRepRluc-WT. The vesicular stomatitis virus G glycoprotein expression vector, pCMV-VSV-G, was obtained from Cell Biolabs (San Diego, CA). pCMV-LAI gp160 was made by inserting gp160-encoding sequences from HIV-1 LAI into a pcDNA expression vector (Thermo Fisher Scientific, Waltham, MA). Synthesis of GSK878 has been described previously (42). Reference compounds, raltegravir (RAL), efavirenz (EFV), nelfinavir (NFV), and lenacapavir (LEN), were synthesized at ViiV. Cyclosporine (CsA) was purchased from Sigma-Aldrich (St. Louis, MO).

### Drug susceptibility assays.

NLRepRluc virus stocks were generated by transfection of pNLRepRluc DNA clones into HEK-293T cells using TransIT-293 transfection reagent (Mirus Bio, Madison, WI). Virus-containing cell culture supernatants were collected ~54 h posttransfection, clarified by centrifugation (1,000 × *g*, 10 min), aliquoted, and stored at −80°C until needed. NLRepRlucΔEnv pseudovirus stocks were generated by cotransfection of HEK-293T cells with pNLRepRlucΔEnv and an ENV expression vector (pCMV-VSV-G or pCMV-LAI gp160) in a 2:1 ratio. For standard drug susceptibility assays, MT-2 cells were infected with an NLRepRluc virus. Briefly, MT-2 cells were pelleted, resuspended in ~300 μL of complete RPMI 1640 medium without phenol red, and mixed with virus by gentle pipetting. Following incubation at 37°C (~10 min), additional medium was added, and cells were distributed (26,000 cells/well, 200 μL) to 96-well plates (number 3904; Corning, Tewksbury, MA) preseeded with serial dilutions of inhibitors in DMSO (2 μL/well, 3-fold dilutions, columns 1 to 10). A no-inhibition control was obtained from wells plated with infected cells plus 2 μL of DMSO (column 11). Background was derived from wells containing cell culture medium plus 2 μL of DMSO (column 12). Assay plates were incubated at 37°C and 5% CO_2_ for ~96 h, at which time *Renilla* luciferase activity was measured with Enduren reagent (Promega Corp., Madison, WI). Relative light units (RLUs) were measured with an EnVision multilabel plate reader (PerkinElmer, Inc., Waltham, MA). Background was subtracted from all wells, and percent inhibition was calculated relative to the value for DMSO-treated control cells (mean RLUs from column 11). In all assays, serial dilutions of inhibitors were plated in duplicate and RLU values from replicate wells were averaged. Virus inocula yielding ~100,000 RLUs/well in DMSO-treated control cells were used for the drug susceptibility assays with replicating NLRepRluc viruses. VSV-G:NLRepRlucΔEnv virus inocula yielding ~10,000 to 20,000 RLUs were used for single-cycle pseudovirus assays. Assays with full-length HIV-1 in A3R5-GFP/Luc cells were performed as described for the NLRepRluc reporter viruses, with the following differences: (i) assays were performed with 20,000 cells per well, (ii) background was obtained from uninfected cells plated in column 12 with 1% DMSO, and (iii) firefly luciferase expression from the integrated reporter gene was measured with a Bright-Glo luciferase assay system (Promega Corp., Madison, WI). For cytotoxicity assays, uninfected cells were plated with serial dilutions of inhibitor as described for the drug susceptibility assays. After 4 days of incubation, cell viability was measured with XTT [2,3-bis-(2-methoxy-4-nitro-5-sulfophenyl)-2H-tetrazolium-5-carboxanilide salt] reagent (Alpha Aesar, Tewksbury, MA). EC_50_ and EC_90_ values were calculated with a log inhibitor-versus-response, variable-slope model [Y = bottom + (top-bottom)/(1 + 10^((logEC50-X) × Hillslope)^)] (GraphPad 8.0 or E-WorkBook software; ID Business Solutions Ltd., Guildford, UK). Unpaired *t* tests (GraphPad 8.0) were used to evaluate the statistical significance between GSK878 effective concentrations toward NLRepRluc-WT and NLRepRluc-H87P viruses.

### Assay to determine early versus late antiviral activity.

HEK-293T cells (~80% confluent) in 75-cm^2^ CellBind tissue culture flasks (Sigma-Aldrich, St. Louis, MO) were cotransfected with pNLRepRlucΔEnv and pCMV-LAI gp160 vectors (~2:1 ratio) by using TransIT-293 transfection (Mirus Bio, Madison, WI). After 4 h, cells were collected by pipetting and transferred (50,000 cells/well) to 96-well BioCoat cell culture plates (Thermo Fisher Scientific, Waltham, MA) in the presence (late-phase inhibition) or absence (early-phase inhibition) of serially diluted inhibitors in DMSO (10 concentrations; 3-fold dilutions). Three days posttransfection, cell culture supernatants were collected and transferred (2 μL/well) to 96-well assay plates containing B6 reporter cells (20,000 cells/well) with (early-phase inhibition) or without (late-phase inhibition) serial dilutions of inhibitors (10 concentrations; 3-fold dilutions in triplicate). A 1% DMSO concentration was maintained in all wells throughout the experiment. After 72 h, firefly luciferase expression from the B6 reporter cells was measured by using Bright-Glo luciferase reagent (Promega Corp., Madison, WI). Percent inhibition relative to that of DMSO-treated control cells was used to calculate EC_50_ values by using GraphPad Prism 8.0 software (ID Business Solutions, Ltd., Guildford, UK). For p24 analysis, HEK-293T cells were cotransfected with pNLRepRlucΔEnv and pCMV-LAI gp160 vectors. After 4 h, cells (50,000/well) were transferred to 96-well assay plates with serial dilutions of inhibitors (10 concentrations; 3-fold dilutions). After 72 h, CA antigen (p24) in medium from transfected HEK-293T cells was quantified by using an HIV p24 (high sensitivity) AlphaLISA detection kit following the manufacturer’s protocol (PerkinElmer, Inc., Waltham, MA). The concentration of p24 antigen relative to that of DMSO-treated control cells was used to calculate half-maximal inhibitory concentration (IC_50_) values (GraphPad Prism 8.0; ID Business Solutions, Ltd., Guildford, UK).

### Time-of-addition assay.

MT-2 cells were infected with NLRepRlucΔEnv virus pseudotyped with HIV-1 LAI gp160 as described for drug susceptibility assays. Following infection, cells were resuspended in complete RPMI 1640 medium without phenol red containing 0.5% DMSO and distributed to 96-well assay plates (26,000 cells/well in 200 μL). Inhibitors (1 μL in DMSO) or DMSO (1 μL) was added to cells at the time of plating (*T*_0_) or at 2, 4, 5, 6, 7, or 8 h after infection with 8 replicate wells per compound. Luciferase activity was measured 72 h postinfection with Enduren reagent (Promega Corp., Madison, WI). Background (mean RLUs from wells containing culture medium plus DMSO without cells) was subtracted from all wells. Percent inhibition was calculated relative to the value for DMSO-treated control cells.

### Quantitative PCR (qPCR) assays.

NLRepRlucΔEnv virus pseudotyped with VSV-G was produced by transfection of HEK-293T cells as described above. Harvested virus was cleared by centrifugation and passed through a 0.45-μm Millex-HV filter (MilliporeSigma, Burlington, MA). Aliquots were store at −80°C until needed. Before infection, virus aliquots were treated with DNase I (10 U/mL; Thermo Fisher Scientific, Waltham, MA) to remove residual transfected DNA. The pellet infection method (see “Drug susceptibility assays” above) was used to infect 2 × 10^6^ MT-2 cells with the VSV-G:NLRepRlucΔEnv virus. Infected cells were washed 3 times with complete medium (RPMI 1640 medium without phenol red supplemented with 10% FBS, 10 mM HEPES, 2 mM l-glutamine, 100 U/mL of penicillin G, and 100 μg/mL of streptomycin), resuspended in 10 mL of complete medium, and placed in a T-25 tissue culture flask. Inhibitors or DMSO (no-inhibitor control; 0.1% [vol/vol]) was added and the cells were cultured for 12, 24, or 48 h for quantification of total reverse transcripts, 2-LTR circles, and integrated proviruses, respectively. At the indicated times, total cellular DNA was extracted by using a DNeasy blood and tissue kit (Qiagen, Valencia, CA). For analysis of total reverse transcripts and 2-LTR circles, DNA samples were treated with DpnI (0.2 U/μL; Thermo Fisher Scientific, Waltham, MA) to remove any methylated DNA carried over from vector (pNLRepRlucΔEnv) transfection.

qPCRs for total reverse transcripts and 2-LTR circles were performed as previously described (56), with modifications as noted below. Primers and probe for the total reverse transcript qPCR assay were MH531-5′-TGTGTGCCCGTCTGTTGTGT-3′ (forward primer), MH532-5′-GAGTCCTGCGTCGAGAGATC-3′ (reverse primer), and 5′-6-carboxyfluorescein (FAM)-CAGTGGCGCCCGAACAGGGA-6-carboxytetramethylrhodamine (TAMRA)-3′ (probe). Primers for the 2-LTR circle qPCR assay were MH535-5′-AACTAGGGAACCCACTGCTTAAG-3′ (forward primer) and MH536-5′-TCCACAGATCAAGGATATCTTGTC-3′ (reverse primer). These were used in combination with a probe that spanned the 2-LTR circle junction site, 5′-FAM-CTCTAGCAGTACTGGAAGGGCTA-TAMRA-3′ (84). qPCRs for total reverse transcripts and 2-LTR circles were assembled by mixing DNA (~4% of total cellular DNA for RT and 8% for 2-LTR circles), primers (300 nM), probe (100 nM), and TaqMan fast advanced master mix in 20-μL volumes per the manufacturer’s protocol (Thermo Fisher Scientific, Waltham, MA). Reactions were performed on a QuantStudio 6 Flex system using Real-Time PCR software v1.3 (Applied Biosystems, Waltham, MA). The reaction program consisted of 2 min at 50°C and 2 min at 95°C, followed by 40 cycles of 95°C for 1 s and 60°C for 20 s. Standard curves for the assays were obtained from parallel reactions performed in 8-point triplicate using 1:4 serial dilutions of control amplicons (977 to 4 × 10^6^ copies) plus a no-DNA control with total reverse transcript and 2-LTR amplicon standard controls. All sample values were normalized to glyceraldehyde-3-phosphate dehydrogenase (GAPDH) by the addition of human GAPDH endogenous control probe (VIC/MGB probe; Primer Limited, Thermo Fisher Scientific).

Integrated HIV-1 provirus was measured using a two-step Alu-PCR method (85). An initial amplification step was performed with an Alu-specific primer and an HIV Gag primer. Reactions (50 μL) contained 12.5 ng of sample DNA, 100 nM Alu forward primer (5′-GCCTCCCAAAGTGCTGGGATTACAG-3′), 600 nM HIV-1 Gag reverse primer (5′-GCTCTCGCACCCATCTCTCTCC-3′), and 5 U of Platinum *Taq* DNA polymerase (Thermo Fisher Scientific, Waltham, MA). An integrated HIV-1 DNA control sample (293T long-term-infected [LTI] HIV-1 DNA, a generous gift from F. Bushman) was used to derive a standard curve. The 293T LTI HIV-1 integrated DNA standard was diluted with total cellular DNA from MT-2 cells and used as a template in duplicate PCRs at concentrations ranging from 0.0061 to 25 ng (1:4 serial dilutions) together with an MT-2 cell DNA-only control. A second set of reactions were carried out with the HIV-1 Gag primer only. The first-round PCR program consisted of 95°C for 2 min, followed by 20 cycles of 95°C for 15 s, 50°C for 15 s, and 72°C for 2 min 30 s. To convert the LTI HIV-1 integrated DNA reactions to copy number, 10-μL volumes of the first-round PCR products were amplified in parallel reactions with serial dilutions of the HIV cDNA total transcript standard as described above (total reverse transcript analysis), providing a standard curve for the second-round qPCRs. Reactions were run in duplicate and the mixtures contained 300 nM HIV-1 LTR forward primer (5′-GCCTCAATAAAGCTTGCCTTGA-3′), 300 nM HIV-1 LTR reverse primer (5′-TCCACACTGACTAAAAGGGTCTGA-3′), 100 nM LTR molecular beacon probe {FAM-GCGAGTGCCCGTCTGTTGTGTGACTCTGGTAACTAGCTCGC-DABCYL [4-((4-(dimethylamino)phenyl)azo)benzoic acid]}, and TaqMan fast advanced master mix. The qPCR program was the same as described above for the total reverse transcript and 2-LTR circle amplification. The second-round qPCR was then performed in duplicate with 10 μL of the first-round PCR samples (including 293T LTI HIV-1 integrated DNA standard), 250 nM HIV-1 LTR forward primer, 250 nM HIV-1 LTR reverse primer, 200 nM LTR molecular beacon probe, and TaqMan fast advanced master mix. The final integrated provirus copy number for each sample was obtained by subtracting the copy number obtained from the HIV Gag-only primer amplification from the Alu forward primer-HIV Gag reverse primer amplification copy number. Copy numbers for total reverse transcripts, 2-LTR circles, and integrated provirus were determined as percent relative to the DMSO control.

### Fate-of-the-capsid assay.

The fate-of-the-capsid assay (61, 77) was performed as follows. HeLa cells were plated in 6-well plates (10^6^ cells/well) 1 day prior infection. Spinoculation was carried out by replacing the cell culture medium with 2.5 mL of medium containing VSV-G:NLRepRlucΔEnv virus (~110 μg/mL of p24), followed by centrifugation (2,600 rpm, 30°C, and 2 h). After centrifugation, the cell culture supernatant was replaced with medium (2.5 mL) containing inhibitor or DMSO. Plates were incubated at 37°C for 6 h, at which time cells were harvested in medium containing pronase (3.5 mg/mL; Sigma-Aldrich, St. Louis, MO), collected by centrifugation, and washed with cold phosphate-buffered saline (PBS; 3×). The cell pellets were lysed in 650 μL of hypotonic buffer (10 mM Tris-HCl [pH 8.0], 10 mM KCl, and 1 mM EDTA [pH 8.0]; Pierce protease inhibitor cocktail) and homogenized by passing through a Qiashredder column (Qiagen, Valencia, CA). The cell culture lysate was collected and 50 μL was reserved for later analysis (input). The remaining lysate was layered on the top of a 50% (wt/wt) sucrose cushion in polycarbonate centrifuge tubes (number 349622; Beckman Coulter, Indianapolis, IN) and centrifuged using a TLA-110 fixed-angle rotor (Beckman Coulter) at 60,000 rpm (~100,000 × *g*) at 4°C for 2 h. After centrifugation, 200 μL of supernatant was harvested from the top as the soluble fraction. The sucrose cushion was aspirated, and the pelleted fraction was collected in 30 μL of hypotonic buffer. Input and soluble and pellet fractions (3 μL each) were separately analyzed and quantified by Simple Western automated Western blotting following the manufacturer’s protocol (SimpleProtein Wes; Bio-Techne, Minneapolis, MN). CA was detected with HIV-1 p55 plus p24 plus p17 rabbit polyclonal antibody (1:1,000; Abcam; ab63917).
