# Supplementary material for: Antiviral Properties of HIV-1 Capsid Inhibitor GSK878
Source: Antimicrob Agents Chemother. 2023 Apr 11;67(5):e01694-22. doi: 10.1128/aac.01694-22 (PMC10190262; doi:10.1128/aac.01694-22)
Supplement: Supplemental file 1 — Supplemental material. Download aac.01694-22-s0001.pdf, PDF file, 0.6 MB [file aac.01694-22-s0001.pdf]

Supplemental Table 1. Susceptibility of chimeric reporter viruses to GSK878 and RAL

	GSK878		RAL	
Virus	EC_{50} nM ± SD	N	EC_{50} nM ± SD	N
92UG029-A	0.153 ± 0.022	6	2.49 ± 0.71	8
93TH051-AE	0.057 ± 0.028	6	1.41 ± 0.51	8
93TH062-AE	0.078 ± 0.017	6	2.80 ± 0.67	8
2000166-B	0.034 ± 0.021	4	1.84 ± 1.03	6
4345-B	0.061 ± 0.007	4	2.82 ± 0.47	6
91US005-B	0.093 ± 0.020	4	5.18 ± 2.17	6
91US056-B	0.071 ± 0.007	4	2.65 ± 0.68	6
92BR003-B	0.099 ± 0.018	4	3.84 ± 0.70	6
92BR004-B	0.047 ± 0.011	4	2.72 ± 0.57	6
92BR014-B	0.080 ± 0.020	4	2.67 ± 1.45	6
92BR020-B	0.067 ± 0.028	4	5.24 ± 4.65	6
92BR026-B	0.037 ± 0.006	4	2.27 ± 0.77	6
92BR028-B	0.113 ± 0.027	4	3.81 ± 2.35	8
92TH026-B	0.032 ± 0.009	4	2.37 ± 1.62	6
92US660-B	0.038 ± 0.007	4	2.37 ± 1.18	6
92US712-B	0.067 ± 0.021	4	2.46 ± 0.80	8
92US714-B	0.090 ± 0.011	4	3.13 ± 1.31	6
92US715-B	0.028 ± 0.006	4	2.29 ± 0.79	6
93BR012-B	0.044 ± 0.017	6	2.85 ± 0.40	8
93BR013-B	0.070 ± 0.004	4	3.89 ± 0.74	6
93BR015-B	0.070 ± 0.027	4	2.16 ± 0.72	6
93BR017-B	0.116 ± 0.021	4	3.05 ± 1.24	6
93BR021-B	0.102 ± 0.018	4	3.18 ± 1.30	6
93US074-B	0.034 ± 0.017	4	1.75 ± 0.99	6
93US141-B	0.041 ± 0.023	4	2.70 ± 0.42	6
93US149-B	0.050 ± 0.010	4	2.52 ± 1.09	6
ASM57-B	0.048 ± 0.015	4	2.48 ± 0.94	6
BRH34807-B	0.065 ± 0.008	4	3.73 ± 2.12	6
91USBMS4-B	0.054 ± 0.014	4	2.16 ± 0.78	6
10215-6-C	0.099 ± 0.011	4	1.82 ± 0.46	6
11398-4-C	0.126 ± 0.029	4	2.93 ± 1.30	6
20635-4-C	0.127 ± 0.016	4	2.29 ± 0.42	6
20706-3-C	0.124 ± 0.016	4	2.31 ± 0.82	6
20887-C	0.105 ± 0.014	4	2.25 ± 0.85	6
21068-C	0.170 ± 0.029	4	2.68 ± 0.77	14
92IN101-C	0.083 ± 0.011	4	2.18 ± 0.52	6
92RW026-C	0.098 ± 0.038	4	2.65 ± 0.51	6
93MW595-C	0.124 ± 0.027	4	3.69 ± 1.52	16
97ZA003-C	0.143 ± 0.058	4	2.99 ± 1.33	12
97ZA009-C	0.201 ± 0.042	8	2.70 ± 1.16	22
98CN006-C	0.118 ± 0.029	4	3.17 ± 0.70	8
98IN026-C	0.102 ± 0.023	4	3.09 ± 0.71	6
98TZ017-C	0.090 ± 0.020	4	2.50 ± 0.53	6
MJ4-C	0.130 ± 0.006	4	2.91 ± 0.33	11
pC31-8-C	0.185 ± 0.051	6	2.49 ± 0.88	14
pC40-5-C	0.251 ± 0.022	6	1.49 ± 0.46	8
93BR020-F	0.144 ± 0.037	6	3.53 ± 1.09	8
G3-G	0.158 ± 0.071	6	2.39 ± 1.17	6
Mean (48 viruses)	0.094 ± 0.049		2.77 ± 0.77	

NLRepRluc viruses with Gag-Pr sequences derived from HIV-1 clinical Isolates. Assayed in MT-2 cells

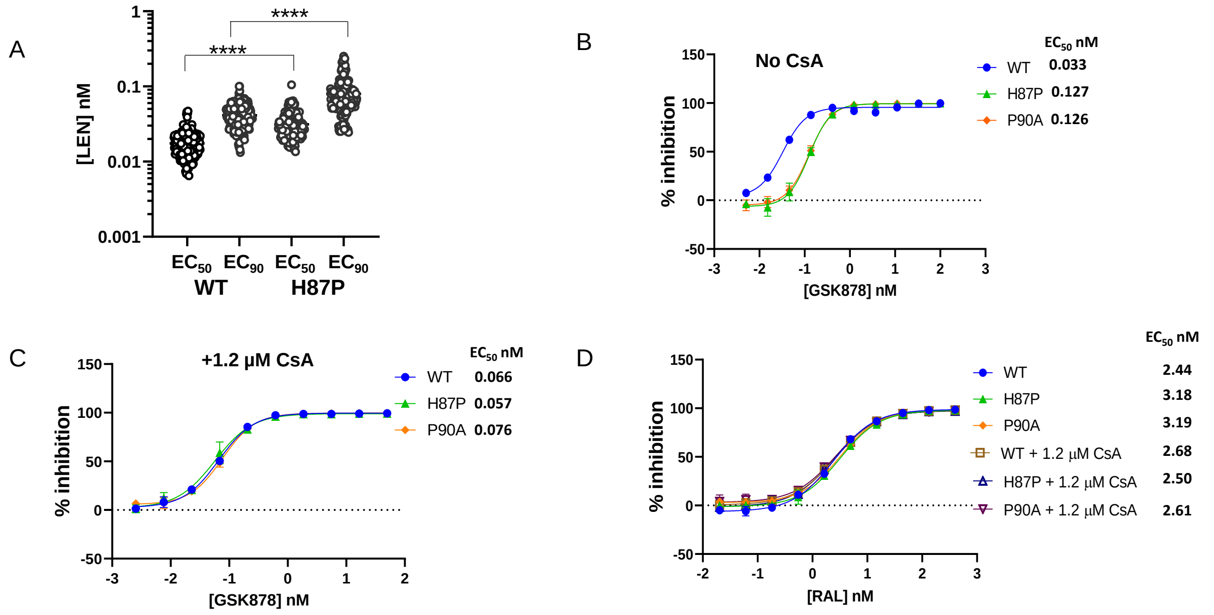

Supplemental Figure 1. Effect of Cyclophilin A on capsid inhibitor antiviral activity. (A) Scatterplot of EC_{50} and EC_{90} values of LEN from antiviral assays with wild-type (WT) and CA H87P HIV-1 reporter viruses (NLRepRluc) in MT-2 cells (****P < 0.0001, WT N=219; H87P N=193). (B) and (C) Comparison of GSK878 dose-response inhibition curves from antiviral assays in MT-2 cells with VSV-G pseudotyped replication defective HIV-1 (VSV-G:NLRepRlucΔENV) with the indicated CA missense mutations in the absence (B) or presence (C) of 1.2 µM CsA. Points are the average of two experiments each performed in duplicate. (D) Dose response curves with a reference INSTI (RAL) from the same conditions in B and C.
